# Association between the coexistence of chronic kidney disease and sarcopenia with cardiovascular disease and mortality

**DOI:** 10.1007/s40520-025-03003-w

**Published:** 2025-03-17

**Authors:** Lijun Jiang, Liangliang Xu, Wen Sun, Keyu Bian, Yuan Wang

**Affiliations:** 1 Department of Nephrology, Wujin TCM Hospital Affiliated to Nanjing University of Chinese Medicine, Changzhou, Jiangsu China; 2 Department of General Practice, Community Health Service Center of Lanling Street, Tianning District, Changzhou City, Jiangsu Province China; 3 Department of Neurology, Centre for Leading Medicine and Advanced Technologies of IHM, The First Affiliated Hospital of USTC, Division of Life Sciences and Medicine, University of Science and Technology of China, Hefei, Anhui China; 4 Department of Neurology, Wujin TCM Hospital Affiliated to Nanjing University of Chinese Medicine, Changzhou, Jiangsu China; 5 Department of Pediatrics, Wujin Six People’s Hospital, Changzhou, Jiangsu China

**Keywords:** Chronic kidney disease, Sarcopenia, Cardiovascular disease, Mortality, Stroke, Heart failure, Coronary artery disease

## Abstract

**Background:**

Chronic kidney disease (CKD) and sarcopenia are independently associated with adverse cardiovascular and mortality outcomes. However, the combined impact of CKD and sarcopenia remains poorly understood. To evaluate the combined effects of CKD and sarcopenia on cardiovascular disease (CVD) and mortality risks in a large population-based cohort.

**Methods:**

We analyzed data from 477,380 participants in the UK Biobank, categorized into four groups based on the presence or absence of CKD and sarcopenia: Non-CKD Non-Sarcopenia, Non-CKD Sarcopenia, CKD Non-Sarcopenia, and CKD Sarcopenia. Cox proportional hazards models estimated hazard ratios (HRs) with 95% confidence intervals (CIs) for CVD and mortality outcomes. Kaplan-Meier survival analyses compared event-free survival across the groups.

**Results:**

Participants with both CKD and sarcopenia exhibited the highest risks across all outcomes compared to those without either condition. For stroke, the adjusted HR was 2.17 (95% CI: 1.65–2.86), significantly higher than CKD alone (HR: 1.69, 95% CI: 1.47–1.94) or sarcopenia alone (HR: 1.28, 95% CI: 1.03–1.59). Similar trends were observed for coronary artery disease (CAD) and heart failure (HF), with HRs of 1.53 (95% CI: 1.38–1.69) and 2.22 (95% CI: 1.99–2.47), respectively, in the CKD-sarcopenia group. The coexistence of CKD and sarcopenia was also associated with significantly elevated all-cause mortality (HR: 2.59, 95% CI: 2.17–3.09) and cardiovascular-specific mortality (HR: 4.08, 95% CI: 2.95–5.66).

**Conclusion:**

The coexistence of CKD and sarcopenia significantly amplifies the risks of CVD and mortality, highlighting the need for integrated management strategies to address this high-risk population. Early detection and tailored interventions targeting these dual risk factors may mitigate their compounded burden and improve clinical outcomes.

**Supplementary Information:**

The online version contains supplementary material available at 10.1007/s40520-025-03003-w.

## Introduction

Cardiovascular disease (CVD) remains a leading cause of morbidity and mortality worldwide, posing a significant public health burden [1]. Chronic kidney disease (CKD) and sarcopenia, two prevalent conditions in aging populations, are individually recognized as key risk factors for adverse health outcomes [2, 3]. CKD is associated with impaired renal function, systemic inflammation, and metabolic disturbances, which significantly elevate the risk of CVD [4]. Similarly, sarcopenia, characterized by progressive loss of skeletal muscle mass and strength, has been linked to increased frailty, reduced functional capacity, and higher susceptibility to chronic illnesses, including CVD [5]. 

Emerging evidence highlights the potential interplay between CKD and sarcopenia. Both conditions share overlapping pathological mechanisms, such as chronic inflammation, oxidative stress, and metabolic dysregulation, which may act synergistically to exacerbate cardiovascular and mortality risks [6, 7]. Despite this, the prevalence of CKD and sarcopenia coexisting in the population remains inadequately explored. Additionally, the combined effects of these two conditions on CVD and mortality outcomes have yet to be fully clarified.

To address these gaps, this study has three primary objectives: (1) to estimate the prevalence of CKD and sarcopenia coexistence in a large population cohort, (2) to evaluate the impact of CKD and sarcopenia on the risk of developing CVD, and (3) to investigate their combined influence on all-cause and cause-specific mortality. By examining these associations, the findings aim to provide novel insights into the compounded burden of CKD and sarcopenia, with potential implications for clinical risk stratification and intervention strategies.

## Method

### Study design and participants

**Fig. 1:**
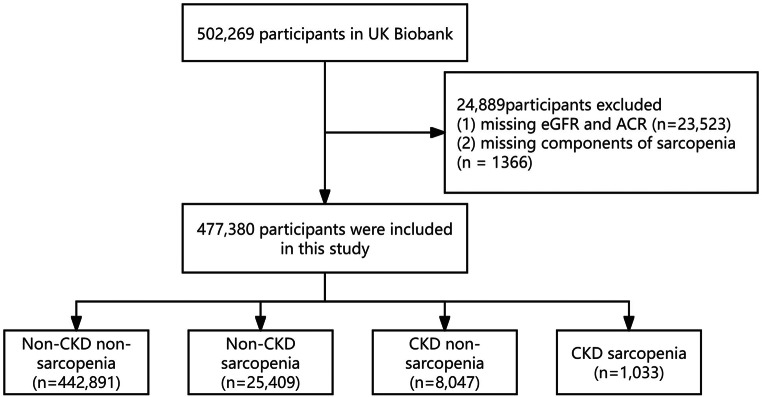
Flow chart

Between 2006 and 2010, approximately 500,000 participants aged 37 to 73 were enrolled in the UK Biobank cohort from across the United Kingdom. Comprehensive data encompassing health, lifestyle, and other variables were collected at baseline and during follow-up assessments through touchscreen questionnaires, verbal interviews, physical measurements, and biometric sampling. Detailed descriptions of the cohort have been published previously [8]. Of these participants, 24,889 were excluded due to missing data on estimated glomerular filtration rate (eGFR) and albumin-to-creatinine ratio (ACR) (*n* = 23,523) or the absence of sarcopenia-related data (*n* = 1,366). Consequently, the final analysis included 477,380 participants (Fig. 1).

### Assessment of sarcopenia

Sarcopenia was defined as the coexistence of low muscle strength and low muscle quantity, following the criteria established by the European Working Group on Sarcopenia in Older People 2 (EWGSOP2) [9]. Muscle strength was assessed using grip strength, calculated as the mean of values recorded for the right and left hands, measured at baseline with a Jamar J00105 hydraulic hand dynamometer. The thresholds for low grip strength were set at < 27 kg for men and < 16 kg for women.

Skeletal muscle mass index (SMI) was determined using bioelectrical impedance analysis (BIA). Specifically, SMI was calculated as appendicular skeletal muscle mass (ASM) divided by height squared. ASM was estimated using the Janssen equation [10], which incorporated resistance values obtained from the Tanita BC418MA body composition analyzer. Low muscle quantity was defined as an SMI < 7.0 kg/m² for men and < 5.5 kg/m² for women, or alternatively, as ASM/Body mass index (BMI), with cutoffs of < 0.55 for women and < 0.84 for men.

### Assessment of chronic kidney disease

Kidney function indices, including serum Estimated Glomerular Filtration Rate (eGFR) and Albumin-to-Creatinine Ratio (ACR), were assessed using the DxC800 synchronous clinical system. The following CKD-EPI formula was employed to calculate eGFR [11]: eGFR = 142 x min(standardized Scr/κ,1) α * max(standardized Scr/κ,1) -1.200 * 0.9938 age in years * 1.012 [if female]. In this formula: Scr represents serum creatinine level. κ is a gender-specific coefficient: 0.7 for females and 0.9 for males.α is -0.241 for females and − 0.302 for males. Both eGFR and ACR were used to diagnose CKD. According to the extended CKD definition, individuals with an eGFR below 60 mL/min/1.73 m² or an ACR exceeding 30 mg/g were classified as having CKD [12]. 

### Outcomes

#### Cardiovascular diseases

CVD include conditions such as stroke, coronary artery disease (CAD), and heart failure (HF). Participants with CVD were identified using the corresponding International Classification of Diseases, 10th edition (ICD-10) codes, specifically stroke (I64), HF (I50), and CAD (I20, I21, I22, I23, I24.1, and I25.2). Identification was achieved through linkage to primary care records, hospital admission electronic health records, and death registry data. The follow-up period was defined as the time from baseline to the earliest occurrence of an incident disease, death, loss to follow-up, or the last follow-up date, whichever occurred first.

#### Mortality

Mortality outcomes included all-cause mortality and CVD mortality. Dates and causes of death were sourced from death certificates provided by the National Health Service (NHS) Information Centre (England and Wales) and the NHS Central Register Scotland (Scotland). Mortality data were updated until October 2023, with follow-up censored at either this date or the date of death, whichever came earlier.

#### Covariates

Participants’ demographic and clinical characteristics, including age, sex, smoking status, drinking status, and comorbidities (e.g., hypertension and hyperlipidemia), were obtained through a self-administered touchscreen questionnaire. Smoking and drinking statuses were classified into three categories: never, former, and current. Socioeconomic deprivation was evaluated using the Townsend Deprivation Index, derived from participants’ postal codes and corresponding national census data, with higher scores indicating greater deprivation [13]. Ethnicity was self-reported and categorized into four groups: White, Black, Asian, and other ethnic backgrounds.Additional lifestyle information included sleep duration, categorized as normal (7–9 h), long (> 9 h), or short (< 7 h). Physical activity levels were measured in metabolic equivalent of task (MET) minutes per week, representing energy expenditure for various activities.Furthermore, fasting blood glucose, glycated hemoglobin (HbA1c), low-density lipoprotein (LDL), and high-density lipoprotein (HDL) levels were also collected for analysis.

### Statistical analyses

Participants were categorized into four groups based on the presence or absence of CKD and sarcopenia: Non-CKD Non-Sarcopenia, Non-CKD Sarcopenia, CKD Non-Sarcopenia, and CKD Sarcopenia. Baseline characteristics were summarized as means with standard deviations (SD) for normally distributed continuous variables, and as medians with interquartile ranges (IQR) for skewed continuous variables. Categorical variables were summarized as frequencies (n) with percentages (%).

A Cox proportional hazards model was employed to investigate the association between the coexistence of CKD and sarcopenia and the risk of incident CVD and mortality. Hazard ratios (HRs) with 95% confidence intervals (CIs) were reported, using the Non-CKD Non-Sarcopenia group as the reference. Model 1: Adjusted for age, sex, ethnicity, and Townsend Deprivation Index. Model 2: Further adjusted for smoking status, alcohol consumption, physical activity (MET hours/week), hypertension, hyperlipidemia, sleep duration, fasting blood glucose, HbA1c, LDL, and HDL. Kaplan-Meier survival analysis was performed to compare event-free survival across the four groups. To reduce potential bias from reverse causality, sensitivity analyses were conducted by excluding participants diagnosed with the outcome of interest within the first two years of follow-up. These sensitivity analyses incorporated the same covariates as the primary models. Statistical significance was defined as a two-sided p-value < 0.05. All statistical analyses and figure generation were conducted using R software (version 4.1.3).

## Results

### Baseline characteristics of the study population

This study included 477,380 participants, divided into four groups: non-CKD non-sarcopenia (*n* = 442,891, 92.9%), non-CKD sarcopenia (*n* = 25,409, 5.3%), CKD non-sarcopenia (*n* = 8,047,1.7%), and CKD sarcopenia (*n* = 1,033, 0.2%). The flow chart was presented in Fig. 1. Baseline Characteristics of the Study Population was shown in Table 1. Participants in the CKD sarcopenia group were older (median age: 65.0 years) and had a higher proportion of males (59.6%) compared to other groups (*P* < 0.001). Hypertension and high cholesterol were more prevalent in the CKD sarcopenia group (73% and 30.6%, respectively) compared to the non-CKD non-sarcopenia group (25.2% and 11.3%, *P* < 0.001). Participants with CKD sarcopenia exhibited the lowest physical activity levels (median MET: 15.9) and the poorest renal function (median eGFR: 53.82 mL/min). Metabolic markers, including fasting blood glucose and HbA1c, were highest in CKD sarcopenia group (*P* < 0.001).

**Table 1:** Baseline characteristics of the subjects

Characteristics	All	Non-CKD non-sarcopenia	CKD non-sarcopenia	Non-CKD sarcopenia	CKD sarcopenia	*P*
Sample size	477,380	442,891 (92.9%)	8047 (1.7%)	25,409 (5.3%)	1033 (0.2%)	
Age (median (IQR))	58.00 [50.00, 63.00]	57.00 [50.00, 63.00]	63.00 [58.00, 67.00]	62.00 [57.00, 66.00]	65.00 [61.00, 68.00]	< 0.001
Male, n (%)	218,851 (45.8)	201,943 (45.6)	3823 (47.5)	12,469 (49.1)	616 (59.6)	< 0.001
Ethnicity, n (%)						< 0.001
White	454,139 (95.2)	422,758 (95.5)	7590 (94.5)	22,869 (90.3)	922 (89.3)	
Black	11,486 (2.4)	9578 (2.2)	218 (2.7)	1619 (6.4)	71 (6.9)	
Asian	3273 (0.7)	2939 (0.7)	91 (1.1)	234 (0.9)	9 (0.9)	
Other	8002 (1.7)	7220 (1.6)	134 (1.7)	617 (2.4)	31 (3.0)	
Hypertension, n (%)	127,388 (26.7)	111,734 (25.2)	4737 (58.9)	10,163 (40.0)	754 (73.0)	< 0.001
High cholesterol, n (%)	57,022 (11.9)	49,872 (11.3)	1973 (24.5)	4861 (19.1)	316 (30.6)	< 0.001
Townsend deprivation index (median (IQR))	-2.14 [-3.65, 0.53]	-2.20 [-3.67, 0.42]	-1.83 [-3.44, 1.16]	-1.17 [-3.14, 2.12]	-0.47 [-2.85, 3.17]	< 0.001
Smoking (%)						< 0.001
Never	259,760 (54.7)	242,744 (55.1)	3832 (48.0)	12,748 (50.7)	436 (42.7)	
Former	164,982 (34.7)	151,762 (34.4)	3344 (41.9)	9404 (37.4)	472 (46.3)	
Current	50,246 (10.6)	46,313 (10.5)	809 (10.1)	3012 (12.0)	112 (11.0)	
Alcohol status, n (%)						< 0.001
Never	21,055 (4.4)	18,296 (4.1)	555 (6.9)	2092 (8.3)	112 (10.9)	
Former	17,147 (3.6)	15,015 (3.4)	488 (6.1)	1545 (6.1)	99 (9.6)	
Current	438,017 (92.0)	408,607 (92.5)	6973 (87.0)	21,621 (85.6)	816 (79.5)	
Sleep time, n (%)						< 0.001
Short	117,134 (24.7)	107,901 (24.5)	1951 (24.6)	6993 (28.0)	289 (28.5)	
Normal	347,920 (73.4)	324,717 (73.8)	5663 (71.3)	16,907 (67.7)	633 (62.5)	
Long	8741 (1.8)	7235 (1.6)	330 (4.2)	1085 (4.3)	91 (9.0)	
MET h/week, median [IQR]	29.77 [13.58, 59.50]	30.22 [13.90, 59.77]	25.77 [10.79, 54.20]	23.62 [9.62, 52.09]	15.90 [5.78, 42.00]	< 0.001
Body mass index, kg/m2	26.76 [24.15, 29.92]	26.65 [24.15, 29.68]	28.36 [25.48, 31.91]	29.29 [23.14, 34.03]	32.02 [27.18, 37.02]	< 0.001
FBG	4.93 [4.60, 5.32]	4.92 [4.60, 5.30]	5.10 [4.70, 5.67]	5.01 [4.65, 5.50]	5.22 [4.69, 6.01]	< 0.001
HbA1c	35.20 [32.80, 37.90]	35.10 [32.70, 37.70]	37.30 [34.40, 41.20]	36.70 [34.00, 40.00]	39.60 [36.00, 46.58]	< 0.001
eGFR (mL/min)	97.26 [87.10, 103.77]	97.50 [87.78, 103.99]	55.79 [49.52, 59.23]	96.90 [87.38, 102.26]	53.82 [46.34, 58.83]	< 0.001
HDL	2.95 [2.57, 3.41]	2.96 [2.57, 3.42]	2.79 [2.39, 3.27]	2.90 [2.52, 3.35]	2.62 [2.29, 3.01]	< 0.001
LDL	2.47 [2.09, 2.88]	2.48 [2.10, 2.89]	2.26 [1.85, 2.72]	2.40 [1.99, 2.84]	2.10 [1.68, 2.53]	< 0.001

### Association between the coexistence of CKD and sarcopenia and CVD

The coexistence of CKD and sarcopenia was associated with significantly higher risks of stroke, CAD, and HF compared to having either condition alone.

**Fig. 2:**
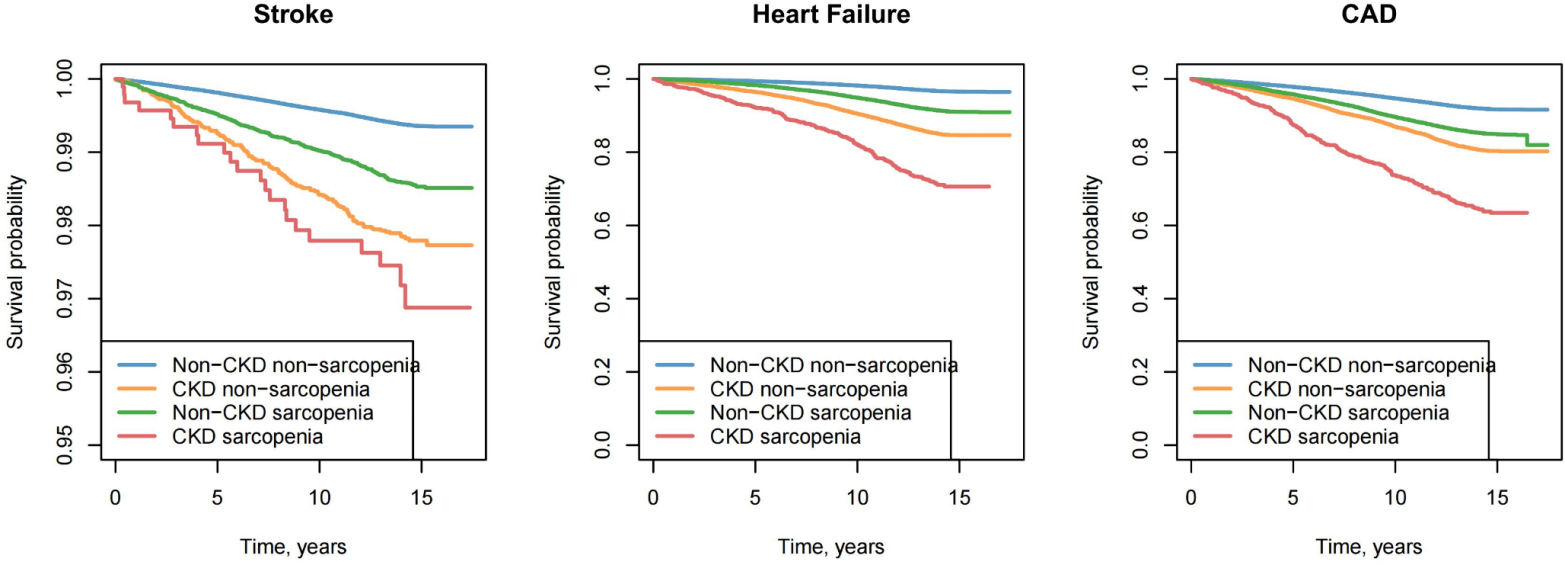
KM survival analysis for cardiovascular diseases

For stroke, the adjusted HR in the CKD sarcopenia group was 2.17 (95% CI: 1.65–2.86), notably higher than the HRs for CKD alone (1.69, 95% CI: 1.47–1.94) and sarcopenia alone (1.28, 95% CI: 1.03–1.59). Similar trends were observed for CAD, with an adjusted HR of 1.53 (95% CI: 1.38–1.69) in the CKD sarcopenia group, compared to 1.43 (95% CI: 1.09–1.89) for CKD alone and 1.17 (95% CI: 1.1–1.26) for sarcopenia alone. The risk of HF was particularly pronounced in the CKD sarcopenia group, with an adjusted HR of 2.22 (95% CI: 1.99–2.47), surpassing the HRs for CKD alone (2.09, 95% CI: 1.61–2.7) and sarcopenia alone (1.25, 95% CI: 1.15–1.37).(Table 2). Kaplan-Meier survival analyses illustrating the association between the coexistence of CKD and sarcopenia and the risk of incident CVD are presented in Fig. 2.

**Table 2:** The association between the coexistence of CKD and sarcopenia and cardiovascular disease

	Stroke		HF		CAD	
	HR (95%CI)	P	HR (95%CI)	P	HR (95%CI)	P
Model 1						
Non-CKD non-sarcopenia	Ref		Ref		Ref	
CKD non-sarcopenia	2.29(1.95–2.71)	< 0.001	3.03(2.85–3.23)	< 0.001	1.88(1.77–1.99)	< 0.001
Non-CKD sarcopenia	1.5(1.33–1.68)	< 0.001	1.64(1.57–1.72)	< 0.001	1.37(1.32–1.42)	< 0.001
CKD sarcopenia	2.41(1.58–3.67)	< 0.001	4.82(4.22–5.5)	< 0.001	3.1(2.73–3.53)	< 0.001
Model 2						
Non-CKD non-sarcopenia	Ref		Ref		Ref	
CKD non-sarcopeni	1.69(1.47–1.94)	< 0.001	2.09(1.61–2.7)	< 0.001	1.43(1.09–1.89)	< 0.001
Non-CKD sarcopenia	1.28(1.03–1.59)	0.027	1.25(1.15–1.37)	< 0.001	1.17(1.1–1.26)	< 0.001
CKD sarcopenia	2.17(1.65–2.86)	< 0.001	2.22(1.99–2.47)	< 0.001	1.53(1.38–1.69)	0.011

### Association between the coexistence of CKD and sarcopenia and mortality

The coexistence of CKD and sarcopenia was also strongly associated with increased all-cause and CVD mortality. Participants with CKD and sarcopenia exhibited the highest all-cause mortality risk, with an HR of 3.74 (95% CI: 3.42–4.1) in Model 1 and 2.59 (95% CI: 2.17–3.09) in Model 2 (Table 3).

**Table 3:** The association between the coexistence of CKD and sarcopenia and mortality

	All-cause mortality		CVD mortality	
	HR (95%CI)	P	HR (95%CI)	P
Model 1				
Non-CKD non-sarcopenia	Ref		Ref	
CKD non-sarcopenia	2.31(2.21–2.41)	< 0.001	3.27(2.98–3.59)	< 0.001
Non-CKD sarcopenia	1.51(1.46–1.56)	< 0.001	1.78(1.66–1.9)	< 0.001
CKD sarcopenia	3.74(3.42–4.1)	< 0.001	6.39(5.37–7.59)	< 0.001
Model 2				
Non-CKD non-sarcopenia	Ref		Ref	
CKD non-sarcopenia	1.97(1.82–2.13)	< 0.001	2.47(2.09–2.92)	< 0.001
Non-CKD sarcopenia	1.38(1.3–1.46)	< 0.001	1.56(1.38–1.77)	< 0.001
CKD sarcopenia	2.59(2.17–3.09)	< 0.001	4.08(2.95–5.66)	< 0.001

**Fig. 3:**
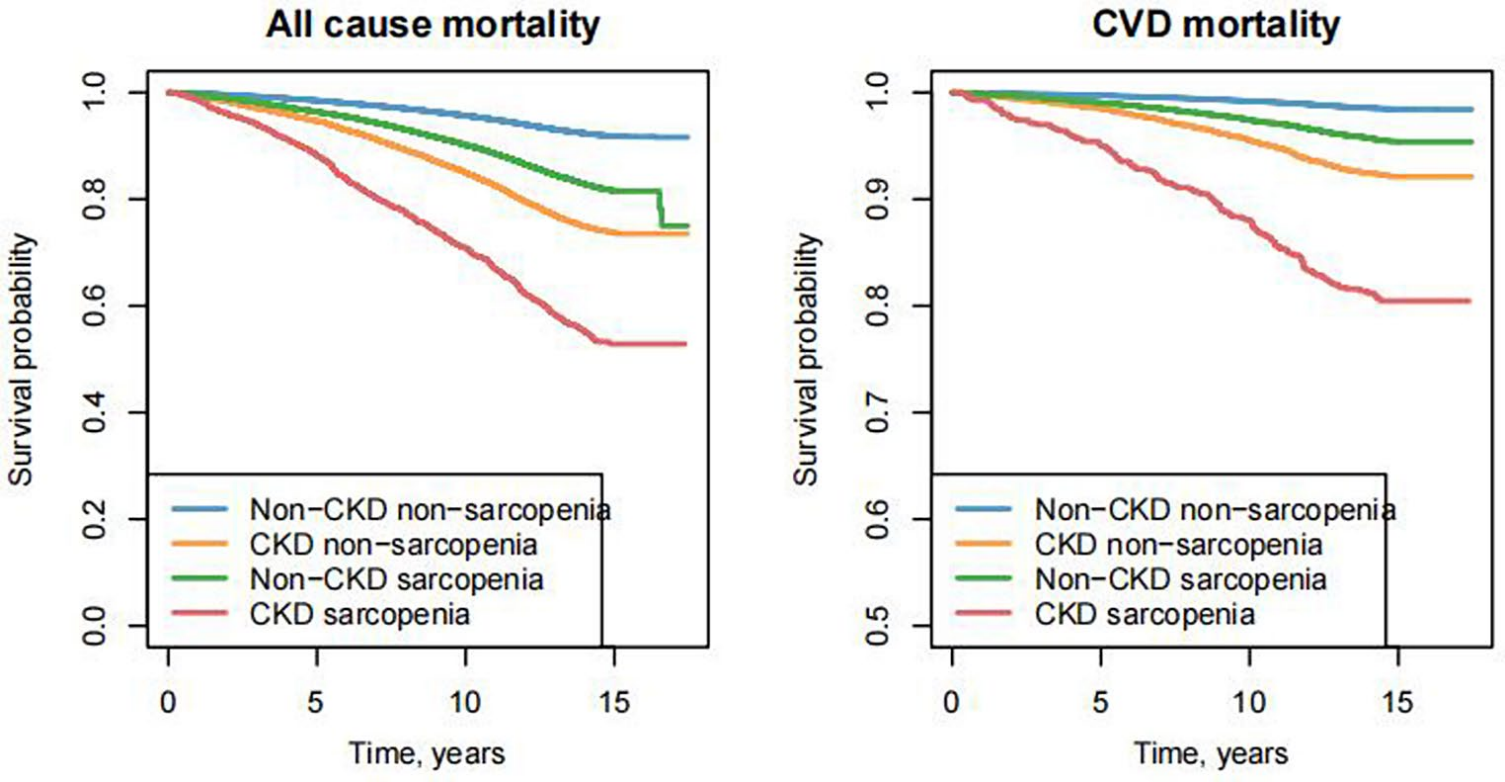
KM survival analysis for all-cause mortality and CVD mortality

Cause-specific analyses further revealed that the combined presence of CKD and sarcopenia led to markedly higher risks of CVD mortality. The HR for CVD mortality in the CKD sarcopenia group was 6.39 (95% CI: 5.37–7.59) in Model 1 and 4.08 (95% CI: 2.95–5.66) in Model 2 (Table 3). Kaplan-Meier survival analyses illustrating the association between the coexistence of CKD and sarcopenia and mortality are presented in Fig. 3.

### Risk of CVD and death associated with sarcopenia in populations with and without CKD

We also analyzed the association between sarcopenia and the risk of incident CVD and mortality in participants with and without CKD. Table S1 presents the risk of CVD and mortality associated with sarcopenia in populations with and without CKD. In both groups, sarcopenia was linked to a significantly increased risk of stroke, CAD, HF, all-cause mortality, and CVD mortality. For individuals without CKD, sarcopenia was associated with a higher risk of stroke (HR 1.49 (95% CI: 1.32–1.67) in Model 1; HR 1.29 (95% CI: 1.04–1.61) in Model 2), CAD (HR 1.36 (95% CI: 1.31–1.42) in Model 1; HR 1.17 (95% CI: 1.1–1.26) in Model 2), HF (HR 1.63 (95% CI: 1.56–1.71) in Model 1; HR 1.24 (95% CI: 1.14–1.35) in Model 2), all-cause mortality (HR 1.5 (95% CI: 1.45–1.55) in Model 1; HR 1.37 (95% CI: 1.29–1.45) in Model 2), and CVD mortality (HR 1.76 (95% CI: 1.65–1.89) in Model 1; HR 1.55 (95% CI: 1.37–1.76) in Model 2). In individuals with CKD, the associations were generally stronger, with higher HRs observed for stroke (HR 1.92 (95% CI: 1.41–2.61) in Model 1; HR 1.54 (95% CI: 1.04–2.29) in Model 2), CAD (HR 1.72 (95% CI: 1.5–1.99) in Model 1; HR 1.28 (95% CI: 1.11–1.48) in Model 2), HF (HR 1.66 (95% CI: 1.43–1.92) in Model 1; HR 1.55 (95% CI: 1.33–1.81) in Model 2), all-cause mortality (HR 1.67 (95% CI: 1.51–1.85) in Model 1; HR 1.4 (95% CI: 1.15–1.71) in Model 2), and CVD mortality (HR 2.05 (95% CI: 1.69–2.49) in Model 1; HR 1.86 (95% CI: 1.28–2.7) in Model 2).

### Sensitivity analysis

The sensitivity analysis displayed in Table S2-3 shows consistent results with the main findings.

## Discussion

This study highlights the significant and synergistic impact of sarcopenia and CKD on CVD and mortality outcomes. By examining a large, well-characterized cohort from the UK Biobank, we demonstrated that the coexistence of sarcopenia and CKD was associated with the risks of stroke, CAD, HF, all-cause mortality, and cardiovascular-specific mortality. These results not only confirm the individual contributions of sarcopenia and CKD to adverse health outcomes but also reveal the compounded burden of their coexistence, emphasizing the importance of recognizing and addressing this dual-risk population in clinical practice.

The strong associations observed in this study are likely driven by overlapping pathophysiological mechanisms. Both sarcopenia and CKD are characterized by heightened systemic inflammation and oxidative stress, which contribute to vascular injury and cardiac remodeling [3, 4]. CKD is associated with elevated pro-inflammatory cytokines, such as IL-6 and TNF-α, and increased oxidative stress due to uremic toxins [4, 14]. Similarly, sarcopenia promotes a pro-inflammatory state, which accelerates endothelial dysfunction, atherosclerosis, and increases cardiovascular risk [15]. CKD exacerbates endothelial dysfunction through impaired nitric oxide production, vascular calcification, and uremic toxins, leading to increased arterial stiffness and hypertension [16, 17]. Sarcopenia, characterized by physical inactivity and metabolic dysfunction, further impairs endothelial function [18]. The reduced physical activity due to sarcopenia limits the benefits of exercise-induced shear stress, compounding the effects of CKD-related vascular impairment. CKD is frequently associated with protein-energy wasting, which accelerates muscle loss and sarcopenia. In turn, sarcopenia worsens CKD progression by reducing physical activity, impairing glucose metabolism, and increasing inflammation [19–21]. This creates a vicious cycle, where CKD exacerbates sarcopenia, and sarcopenia amplifies CKD-related complications, heightening cardiovascular risk [22]. 

Both conditions also contribute to metabolic dysregulation, with CKD altering lipid profiles, increasing insulin resistance, and disturbing calcium-phosphorus balance, while sarcopenia reduces metabolic flexibility, worsening hyperglycemia and metabolic stress [23, 24]. This accelerates cardiovascular complications such as left ventricular hypertrophy and heart failure. Sarcopenia reduces mobility, which exacerbates CKD-related risks by increasing blood pressure, impairing glycemic control, and promoting weight gain. This limits the cardioprotective effects of exercise and accelerates functional decline. Together, sarcopenia and CKD worsen cardiovascular outcomes.

Our findings suggest that the risk of CVD and mortality is higher in individuals with both sarcopenia and CKD compared to those with either condition alone. Participants with both conditions exhibited a hazard ratio for CVD mortality nearly four times higher than those without either condition. These results emphasize the compounded cardiovascular risk in this dual-risk population and highlight the need for targeted interventions.

The differential impact of sarcopenia in populations with and without CKD also warrants further discussion. While sarcopenia was associated with increased risks of CVD and mortality in both groups, the effect was more pronounced in individuals with CKD. This may be explained by the compounded effects of CKD-related metabolic disturbances on muscle health and overall physical function. CKD patients often experience anemia, vitamin D deficiency, and uremic toxins, all of which can accelerate muscle wasting and impair recovery, thereby amplifying the impact of sarcopenia on cardiovascular outcomes [25]. These findings highlight the importance of integrating muscle health assessments into the routine care of CKD patients to identify and manage sarcopenia early.

Our results align with and expand upon existing literature. Previous studies have documented the independent associations of sarcopenia and CKD with CVD and mortality, but few have examined their combined effects. By analyzing a large, diverse cohort with detailed data on health outcomes and covariates, we provide robust evidence of the compounded burden of sarcopenia and CKD. Moreover, the observed associations persisted even after adjusting for key confounders such as age, physical activity, and comorbidities, underscoring the robustness of our findings.

These findings have several practical implications. First, healthcare providers should prioritize the early detection of sarcopenia in CKD patients and vice versa, as addressing both conditions simultaneously may help mitigate their cumulative risks. Interventions such as resistance training, nutritional supplementation, and optimized management of CKD-related complications could play a vital role in improving outcomes. Second, our findings suggest the need for tailored cardiovascular risk prediction models that incorporate both sarcopenia and CKD as key factors. Such models could help identify high-risk individuals and guide the allocation of preventive resources. Finally, public health strategies should focus on promoting healthy aging and addressing the modifiable risk factors for both conditions, such as physical inactivity, poor nutrition, and chronic inflammation.

This study has several limitations. First, its observational design limits the ability to establish causal relationships. While extensive adjustments were made for potential confounders, residual confounding remains possible. Second, sarcopenia was assessed using indirect markers rather than direct measures such as muscle mass or strength, which may lead to misclassification. Third, the study population predominantly consisted of individuals of European ancestry, which may limit the generalizability of the findings to other ethnic groups. Fourth, despite the long follow-up period, unmeasured changes in participants’ health status or interventions during follow-up could influence the outcomes. Lastly, the potential for reverse causality, particularly in the early years of follow-up, cannot be entirely excluded, although sensitivity analyses were performed to address this issue.

## Conclusion

In this study, the coexistence of sarcopenia and CKD was present in 0.2% of the population. The coexistence of sarcopenia and CKD markedly increases the risks of CVD and mortality, with evidence of synergistic effects on adverse outcomes. These findings emphasize the importance of integrated management strategies that address both conditions to reduce their combined burden. Future research should explore the underlying mechanisms driving these interactions and evaluate the efficacy of targeted interventions in mitigating their impact. Enhanced awareness of the compounded risks associated with sarcopenia and CKD could lead to improved risk prediction, personalized care, and better outcomes for affected individuals.

## Electronic supplementary material

Below is the link to the electronic supplementary material.

Supplementary Material 1

## Data Availability

No datasets were generated or analysed during the current study.
