# Supplementary material for: Association between the coexistence of chronic kidney disease and sarcopenia with cardiovascular disease and mortality
Source: Aging Clin Exp Res. 2025 Mar 17;37(1):92. doi: 10.1007/s40520-025-03003-w (PMC11913966; doi:10.1007/s40520-025-03003-w)
Supplement: Supplementary file 1 — Supplementary Material 1 [file 40520_2025_3003_MOESM1_ESM.docx]

Table S1. Risk of CVD and death associated with Sarcopenia in populations with and without CKD.

|  | Without CKD | | With CKD | |
| --- | --- | --- | --- | --- |
|  | HR (95%CI) | P | HR (95%CI) | P |
| stroke |  |  |  |  |
| Model 1 | 1.49(1.32 - 1.67) | <0.001 | 1.92(1.41 - 2.61) | <0.001 |
| Model 2 | 1.29(1.04 - 1.61) | 0.021 | 1.54(1.04 - 2.29) | 0.033 |
| CAD |  |  |  |  |
| Model 1 | 1.36(1.31 - 1.42) | <0.001 | 1.72(1.5 - 1.99) | <0.001 |
| Model 2 | 1.17(1.1 - 1.26) | <0.001 | 1.28(1.11 - 1.48) | 0.001 |
| HF |  |  |  |  |
| Model 1 | 1.63(1.56 - 1.71) | <0.001 | 1.66(1.43 - 1.92) | <0.001 |
| Model 2 | 1.24(1.14 - 1.35) | <0.001 | 1.55(1.33 - 1.81) | <0.001 |
| All-cause mortality |  |  |  |  |
| Model 1 | 1.5(1.45 - 1.55) | <0.001 | 1.67(1.51 - 1.85) | <0.001 |
| Model 2 | 1.37(1.29 - 1.45) | <0.001 | 1.4(1.15 - 1.71) | 0.001 |
| CVD mortality |  |  |  |  |
| Model 1 | 1.76(1.65 - 1.89) | <0.001 | 2.05(1.69 - 2.49) | <0.001 |
| Model 2 | 1.55(1.37 - 1.76) | <0.001 | 1.86(1.28 - 2.7) | 0.001 |

Table S2. Sensitivity analysis excluding events occurring in the first two years.

|  | Stroke |  | HF |  | CAD |  |
| --- | --- | --- | --- | --- | --- | --- |
|  | HR (95%CI) | P | HR (95%CI) | P | HR (95%CI) | P |
| Model 1 |  |  |  |  |  |  |
| Non-CKD non-sarcopenia | Ref |  | Ref |  | Ref |  |
| CKD non-sarcopenia | 2.3(1.93 - 2.74) | <0.001 | 2.91(2.72 - 3.11) | <0.001 | 1.88(1.77 - 2) | <0.001 |
| Non-CKD sarcopenia | 1.46(1.28 - 1.65) | <0.001 | 1.65(1.57 - 1.73) | <0.001 | 1.38(1.33 - 1.44) | <0.001 |
| CKD sarcopenia | 2.3(1.45 - 3.67) | <0.001 | 4.63(4.02 - 5.32) | <0.001 | 3.16(2.76 - 3.62) | <0.001 |
| Model 2 |  |  |  |  |  |  |
| Non-CKD non-sarcopenia | Ref |  | Ref |  | Ref |  |
| CKD non-sarcopenia | 1.66(1.43 - 1.92) | <0.001 | 2.04(1.56 - 2.67) | <0.001 | 1.47(1.1 - 1.96) | <0.001 |
| Non-CKD sarcopenia | 1.31(1.19 - 1.44) | <0.001 | 1.24(1.14 - 1.35) | <0.001 | 1.17(1.09 - 1.26) | <0.001 |
| CKD sarcopenia | 2.15(1.6 - 2.89) | <0.001 | 2.14(1.91 - 2.4) | <0.001 | 1.54(1.38 - 1.71) | 0.01 |

Table S3. Sensitivity analysis excluding patients who died in the first two years.

|  | All-cause mortality |  | CVD mortality |  |
| --- | --- | --- | --- | --- |
|  | HR (95%CI) | P | HR (95%CI) | P |
| Model 1 |  |  |  |  |
| Non-CKD non-sarcopenia | Ref |  | Ref |  |
| CKD non-sarcopenia | 2.28(2.17 - 2.39) | <0.001 | 3.25(2.95 - 3.57) | <0.001 |
| Non-CKD sarcopenia | 1.5(1.45 - 1.55) | <0.001 | 1.77(1.65 - 1.9) | <0.001 |
| CKD sarcopenia | 3.68(3.34 - 4.04) | <0.001 | 6.06(5.04 - 7.28) | <0.001 |
| Model 2 |  |  |  |  |
| Non-CKD non-sarcopenia | Ref |  | Ref |  |
| CKD non-sarcopenia | 1.95(1.79 - 2.11) | <0.001 | 2.49(2.1 - 2.95) | <0.001 |
| Non-CKD sarcopenia | 1.38(1.3 - 1.46) | <0.001 | 1.56(1.37 - 1.78) | <0.001 |
| CKD sarcopenia | 2.5(2.08 - 3.01) | <0.001 | 3.68(2.58 - 5.25) | <0.001 |
